# The role of customer personality in premium banking services

**DOI:** 10.1057/s41264-022-00150-3

**Published:** 2022-03-18

**Authors:** Shirie Pui Shan Ho, Amy Wong

**Affiliations:** 1 UOW College Hong Kong, 1/F Le Billionnaire, Kowloon City, Hong Kong; 2 Singapore University of Social Sciences, 463 Clementi Road, Singapore, 599494 Singapore

**Keywords:** Customer personality, Trust, Satisfaction, Customer loyalty

## Abstract

This paper examines the effects of customer personality, trust, and satisfaction on customer loyalty in premium banking services. Based on a survey of 210 high-net-worth premium banking customers, the study found that the customer personality dimensions of conscientiousness, extroversion, and agreeableness affected trust, while extroversion affected satisfaction. Trust displayed a significant influence on both satisfaction and customer loyalty, while satisfaction partially mediated the effect on customer loyalty via trust. The findings can help managers of affluent banking services understand the important dimensions of customer personality in relational exchanges and develop relevant relationship management strategies to deliver satisfaction and enhance customer loyalty.

## Introduction

In the recent years, the proliferation of financial technology, increased customer sophistication, and commoditisation of banking relationships have disrupted the banking industry. These days, retail banks face a challenging environment as consumers have a plethora of banking, wealth planning, and investment management options from licensed and restricted licensed banks (Yun and Hanson 2020) as well as digital banks (Kaur et al. 2021), which are innovative banking services that are transforming the future of banking with hassle-free, borderless, financial solutions. To differentiate themselves from competition, traditional banks need to pay attention to customer relationship management (CRM) strategies that can be used as a strategic weapon to boost customer satisfaction and retain loyal customers (Gokmenoglu and Amir 2020; Viviani et al. 2021). CRM is especially applicable to retail banks, given the complicated and long-term nature of its business, where relationship development and sustenance is integral in its service delivery (Abrar et al. 2019; Olavarría-Jaraba et al. 2018). Nowadays, most banks provide a full portfolio of wealth management services to high-net-worth premium customers who are often assigned a dedicated relationship manager or a team of expert financial advisors. Through ongoing relational exchanges, close banking relationships with profitable customers can be forged and maintained in the long run, leading to organisational success (Nora 2019; Strandberg et al. 2015). Leveraging on advancement in information technology, CRM tools, strategies, and processes can be aligned to enhance corporate performance (Herman et al. 2020), facilitate customer lifecycle management, and support retail bank capabilities in a favourable manner (Narteh and Braimah 2019). Today, CRM is especially important in the era of COVID-19, which has altered the banking processes and practices in serving high-net-worth customers (Lin et al. 2021).

The concept of CRM draws on the fields of psychology and consumer behaviour, which advocate that consumer personality can play an integral role with regard to the acceptance of CRM strategies (Mishra and Vaithianathan 2015). Scholars have suggested that personality traits influence consumer behaviour and decision making (Mehl et al. 2006; Zillig et al. 2002) in the financial services context (Malvika 2022). As such, personality traits are relevant to the exploration of customer personality in the banking industry (Malvika 2022; Mishra and Vaithianathan 2015). Previous studies have considered the factors leading to customer loyalty (Boonlertvanich 2019; Ji and Prentice 2021; Putra and Putri 2019), yet few have examined the role of customer personality in relational exchanges. The investigation of customer personality attributes can help firms better understand consumer decision making, preferences, and choice sets (Hansen and Sand 2008; Im et al. 2021; Roy et al. 2016; Singh et al. 2020), as customers might select certain brands because of their expression of individual personality, social position, or attainment of specific psychological needs (Vazquez-Carrasco and Foxall 2006). Moreover, Tsao and Hsieh (2012) supported the urgency for further research on personal factors such as personality traits, while Brun et al. (2016) emphasised the need for examining customer characteristics, attitudes, and perceptions of trust, satisfaction, and commitment in retail banks. Firms need to understand the specific service employee actions that can influence customer satisfaction and loyalty (Yun and Hanson 2020) so that personality-targeted relationship management approaches can be implemented to enhance customers’ perceptions of trust and satisfaction, leading to rewarding and pleasurable customer experiences (Mukerjee 2018). Thus, an investigation into the role of customer personality, trust, and satisfaction can promote a better understanding of the management practices and decision making required for effective CRM.

As the link between customer personality and relational outcomes might vary depending on the service context, several scholars have called for further research on personality factors in different cultural contexts and industries (Mishra and Vaithianathan 2015; Mohammad 2015; Purani et al. 2019). Within the psychology literature, the Big Five personality, which is made up of dimensions such as openness to experience, conscientiousness, extroversion, neuroticism, and agreeableness (McCrae and Costa 1987), is a widely adopted framework for understanding personality due to its consistency across culture, time, and age group (Udo-Imeh et al. 2015). To date, it is still the most mainstream and widely accepted framework for examining personality (Udo-Imeh et al. 2015) and is commonly used in the consumer behaviour literature to understand consumer personality traits (Block 1995). Given the reliability and validity of the five dimensions, the Big Five personality is applied to the investigation of customer personality in this study.

Although earlier studies have investigated the links between customer personality attributes and relational outcomes (Bawack et al. 2021; Choi and Hwang 2019; Mishra and Vaithianathan 2015), none has integrated customer personality with trust, satisfaction, and customer loyalty in a single study to ascertain their relationships. In addition, mixed and inconsistent results were found in different studies. For example, Bawach et al. (2021) found that majority of personalities has no significant effect on trust, while Choi and Hwang (2019) reported the positive effect of personality on perceived satisfaction. Jani and Han (2014) and Al-Hawari (2014) verified the personality–satisfaction relationship, while Bove and Mitzifiris (2007) reported non-significant relationships between personality and trust. In view of the mixed results, this paper addresses the gap by examining the effects of the Big Five personality dimensions on trust, satisfaction, and customer loyalty in premium banking services. While prior research showed direct links between Big Five and loyalty (Durukan and Bozaci 2011; Lin 2010), other studies verified its indirect links with satisfaction and/or trust as mediators (Smith 2020; Menidjel et al. 2021). Lin and Worthley (2012) noted that personality exerted a positive influence on consumer emotions, which impacted their satisfaction level and consequently influenced their post-purchase behaviour. Moreover, Smith (2020) confirmed the mediation effect of satisfaction on personality–loyalty relationships. In view of earlier research, this study posits satisfaction as a possible mediator in the relationship between trust and loyalty.

The study contributes to the field of services research by expounding the predictive power of the Big Five personality traits (McCrae and Costa 1987) on the trust–satisfaction–loyalty link. The findings can help managers pinpoint the different customer personality dimensions that can drive trust, satisfaction, and customer loyalty. Focusing on the important dimensions, firms can dedicate their limited resources to improve premium banking service delivery and develop relationship management strategies to retain their key customers.

The paper is organised as follows. The literature review, relevant hypotheses, and conceptual framework are presented. Next, the method and results are discussed. Finally, the paper concludes with theoretical and managerial implications, and suggestions for future research.

## Theoretical background

### Social exchange theory

Social exchange theory (SET), which explains the interaction between two parties during the exchange of resources (Homans 1958), has been widely used to illustrate the relational exchange and reciprocity between individuals (Han et al. 2019; Zhang and Liu 2021). As SET centres around interactions and exchanges between consumers and firms, it is well fitted to explain customer–company relationships, particularly in customer service-oriented contexts (Kim and Qu 2020; Lee et al. 2014). In the consumer behaviour literature, SET is commonly used to explain reciprocity between exchange parties (Jung and Yoo 2019; Zhang and Liu 2021). SET advocates that exchange parties will stay in a relationship as long as there are satisfactory benefits. Most often, individuals choose to enter into a relationship because they expect to capture the maximum amount of satisfaction. In this instance, the satisfaction derived is usually measured as the difference between the individual’s perceived reward and perceived cost.

Within an exchange relationship, feelings of trust can develop depending on the level of mutual reciprocation which happens when one party provides benefit to the other, and the other party returns the benefit at a later stage (Harrigan et al. 2018; Veloutsou 2015). Over a sustained duration of time, these reciprocal behaviours can contribute to the building of trust (Lambe et al. 2001). As foundations of SET, the concepts of trust and satisfaction have been widely examined in the relationship management literature (Hsu et al. 2018) in the banking industry (Liyanaarachchi et al. 2021). Trust can be built via positive social exchanges, ongoing communication, and interpersonal interactions between customers and relationship managers (da Rosa Pulga et al. 2019). Given the importance of SET in understanding relational exchanges in service businesses, this theory formed the underlying basis of the hypothesised model in this study.

### Premium banking services

The service-dominant logic, which advocates the role of customers as value co-creators in reciprocally beneficial service exchanges (Vargo and Lusch 2008), can be applied to the examination of affluent banking service delivery and outcomes (Tam 2019). According to Wirtz and Lovelock (2016), premium banking services encompass frequent physical contact between a customer and a service provider. On the other hand, low contact services entail minimal physical contact between the customer and the service provider. Premium banking services, a type of high contact services, are highly personalised, relational services that require the client’s active participation throughout the service delivery process. Eriksson and Hermansson (2017) accentuated that a customer is considered as a relational customer if he or she had interacted with a designated customer contact employee at least once within a year, hence this study focuses on premium banking services where both the high-net-worth premium customer and relationship manager play an important role in co-creating trust, satisfaction, and customer loyalty through reciprocally beneficial, relational exchanges.

The aim of this study is to examine the effect of the Big Five personality dimensions on trust, satisfaction, and customer loyalty. To investigate the constructs in this study, there is a need for customer relationships to be present. Hence, this study targeted premium banking customers who have established prior relationships with their relationship managers. In the banking industry in Hong Kong, only customers with at least HK$1 million assets are eligible for premium banking services, which includes a dedicated relationship manager who is assigned to look after the full portfolio of the customer, comprising financial and investment needs. These high-net-worth customers are regarded as relational customers instead of transactional customers, as most of them would need to have an existing relationship with their designated relationship manager to achieve the high-net-worth status.

### Customer personality

Personality traits are defined as “an individual’s characteristic and pattern of thought, emotion, and behaviour, together with the psychological mechanisms behind those patterns” (Funder 1997, pp.1–2). Personality traits are archetypes where consumers express themselves and reflect the values, actions, and words of a consumer regarding a product, service, or firm (Kim et al. 2018). As personality traits influence consumer behaviour and decision making, the examination of personality traits is relevant to the study of consumer behaviour and customer personality traits (Malvika 2022; Mehl et al. 2006; Zillig et al. 2002). In the personality literature, there is a consensus regarding the Big Five factors as fundamental dimensions of personality. The Big Five has received considerable support (Weiner and Greene 2008; John and Srivastava 1999) as it is the most widely accepted framework for studying personality (Udo-Imeh et al. 2015).

Within the consumer behaviour literature, prior studies have adopted McCrae and Costa (1987)’s definition to understand consumer personality traits (Block 1995). Given its suitability in understanding consumer behaviour, McCrae and Costa’s Big Five Personality traits are adopted in this study. The five personality dimensions include openness to experience, conscientiousness, extroversion, neuroticism, and agreeableness (Solomon 2018). Openness to experience describes the level which a person is open to new ideas and change, while conscientiousness measures the level of organisation required by an individual and involves traits such as dutiful, planful, and orderly. Extroversion measures how well a person tolerates stimulation from others and captures traits such as outgoing and stimulation oriented, while neuroticism refers to the ability of an individual to cope with stress and reflects emotional reactivity. Agreeableness measures the degree to which an individual defers from others and reflects traits such as affable, friendly, and conciliatory.

### Trust

As an incremental concept of SET, trust is a critical element in various types of service interactions. Trust has been conceptualised in different ways including the willingness to rely on an exchange partner (Moorman et al. 1993), confidence in an exchange partner’s reliability and integrity (Morgan and Hunt 1994), acceptance of vulnerability (Ennew and Sekhon 2007), honesty, confidence, integrity, and trustworthiness (Tabrani et al. 2018), as well as moral obligation (Amin et al. 2013, 2018). In general, consumer trust can be represented by three dimensions, namely the cognitive, affective, and behavioural aspects (Lewis and Weigert 1985). Cognitive trust measures a customer’s confidence in relying on the competence and reliability of the service provider, while affective trust relates to a customer’s confidence in the service provider based on feelings or emotions generated from the care and concern provided by the service provider. Behavioural trust refers to the customer’s action based on the confident expectation that the service provider will act competently and dutifully. As behavioural trust constitutes actions that flow from a state of cognitive and affective trust, it is often treated as an outcome of both cognitive and affective trust (Johnson and Grayson 2005).

Within the financial services industry, trust is commonly discussed from a two-dimensional perspective (Chai et al. 2015; Mostafa et al. 2020); hence, this study adopts a two-dimensional perspective of consumer trust, which consists of cognitive and affective trust. Cognitive and affective trust are highly related as a relationship between exchange parties may start with cognitive trust, and over time, based on accumulated experience, develop into affective trust (McAllister 1995). In a mobile banking context, Mostafa (2020) suggests that trust is a second-order, multidimensional construct with attributes including competence, integrity, and benevolence. Specifically, competence and integrity are regarded as components of cognitive trust, while benevolence is regarded as a component of affective trust. Following Mostafa (2020), this study adopted the definition of trust as a second-order construct comprising cognitive trust (i.e., competence and integrity) and affective trust (i.e., benevolence), which are essential elements in premium banking services.

### Satisfaction

As a fundamental concept of SET, customer satisfaction is a complex and multifaceted construct which consists of numerous factors including customer expectation, emotion (i.e., affective state), cognition (Hansemark and Albinsson 2004; Oliver 1997), accumulation (Brun et al. 2014), and/or transaction (Oliver 1993). Tse and Wilton (1988) referred to customer satisfaction as the difference between the expected performance and the actual performance of a product or service consumption, while Amin (2016) viewed customer satisfaction as a construct that is restricted to transaction-specific judgments that is distinctive from service quality. According to Oliver (1997), customer satisfaction refers to a “consumer’s fulfilment response” towards specific products, services, and experiences, and is a post consumption evaluation that a service provided a pleasing level of consumption that is associated with fulfilment. This definition, which accounted for both the affective and cognitive approaches to customer satisfaction, is adopted in this study.

### Customer loyalty

According to Dick and Basu (1994), customer loyalty to a product, service, or brand must include an affirmative attitude toward the product, service, or brand and a decisive buying behaviour. Customer loyalty comprises of two elements, namely behavioural and attitudinal loyalty. Behavioural loyalty refers to a repeat patronage, where a customer repeatedly buys the exact product or service, while attitudinal loyalty relates to an inclination to stay in a relationship with a firm (Yang and Peterson 2004). As the focus on composite loyalty, which combines behavioural loyalty and attitudinal loyalty, can lead to improved firm profitability (Ennew et al. 2018), this study adopted the combined approach to measure both behavioural and attitudinal loyalty.

## Hypothesis development and conceptual framework

### Customer personality and trust

Previous studies have reported positive relationships between customer personality dimensions and trust (Caliskan 2019; Spake and Megehee 2010; Wang et al. 2018). Webber et al. (2012) found that customer agreeableness is positively related to cognitive and affective trust. In a study conducted to assess the psychometric properties of the propensity to trust scale, a positive link was found between extroversion and trust (Evans and Revelle 2008). Similarly, Hiraishi et al. (2008) found extroversion as a significant predictor of trust in individuals who are high in extroversion, as those individuals prefer to interact with others and develop new relationships based on their general level of trust in others. Further, Siddiqui (2016) validated the impact of personality traits on outcomes such as trust and customer loyalty in a study of mobile phone and credit card services in Pakistan, with agreeableness being the main significant predictor of the Big Five personality dimensions.

Drawing on data from a population survey in Switzerland, Freitag and Bauer (2016) reported that the impact of customer personality dimensions on trust in unfamiliar people is stronger than trust in acquaintances, with conscientiousness and openness to experience being related to both trust in unfamiliar people and acquaintances, whereas agreeableness is related to trust in unfamiliar people only. In a study on e-commence in the COVID-19 pandemic era, Jeon et al. (2021) found that neuroticism was positively related to a consumer’s trust transfer, where highly neurotic consumers were more hesitant in trust transfer compared to consumers who are relatively less neurotic. In another study which investigated the acceptance of automated vehicles in China, Zhang et al. (2020) verified the significant effects of neuroticism and openness on trust. Explicitly, users with an openness to new experience reported higher levels of trust towards automated vehicles, while neurotic users presented a distrustful attitude. Given the findings in the preceding studies on the relationships between customer personality dimensions and trust, we advance the following hypotheses:

**H1a**: Openness to experience is positively related to trust

**H2a**: Conscientiousness is positively related to trust

**H3a**: Extroversion is positively related to trust

**H4a**: Neuroticism is positively related to trust

**H5a**: Agreeableness is positively related to trust

### Customer personality and satisfaction

Previous research has established varying relationships between customer personality and customer satisfaction (Mann and Rawat 2016). Within the retail industry, Castillo (2017) reported positive relationships between the Big Five personality traits, customer empowerment, and customer satisfaction, with openness to experience, conscientiousness, and agreeableness impacting satisfaction with employees through an interactive and consultative selling process. In a survey of undergraduate students, Crawford et al. (2017) found the importance of customer personality in predicting customer satisfaction, while Malik et al. (2018) reported that openness to experience moderated the association between information quality and the satisfaction level of internet users.

Adopting a psychological perspective, Vater and Schröder‐Abé (2015) found that the personality traits of openness to experience, conscientiousness, extroversion, and agreeableness predicted long‐term relationship satisfaction of couples through regulating their positive emotions and engaging in interpersonal behaviours that are affirmative and encouraging, whereas neuroticism is linked to the adjustment of negative emotion and display of detrimental interpersonal behaviour, which can give rise to relationship dissatisfaction. Further, Weidmann et al. (2017) reported significant intrapersonal and interpersonal effects of conscientiousness, neuroticism, and agreeableness on relationship satisfaction. More recently, Patitisa et al. (2021) reported the significant positive effects of Big Five on student satisfaction towards online learning, where students with higher levels of openness, conscientiousness, extraversion, and agreeableness exhibited higher levels of satisfaction. In addition, Moghavvemi et al. (2021) indicated that individuals with openness, conscientiousness, extraversion, and agreeableness experienced higher overall satisfaction in a tourism context, while Kreuzer and Gollwitzer (2021) verified the positive relationship between neuroticism and relationship satisfaction. In view of the supporting literature, we propose the following hypotheses:

#### H1b:

Openness to experience is positively related to satisfaction

#### H2b:

Conscientiousness is positively related to satisfaction

#### H3b:

Extroversion is positively related to satisfaction

#### H4b:

Neuroticism is positively related to satisfaction

#### H5b:

 Agreeableness is positively related to satisfaction

### Trust and satisfaction

Trust and satisfaction are two highly related concepts that are integral to the building of customer relationships. While some researchers argued that satisfaction leads to trust (Bove and Mitzifiris 2007; Konuk 2018; Song et al. 2019), others proposed that trust leads to satisfaction (Arcand et al. 2017; Lainamngern and Sawmong 2019; Omoregie et al. 2019). The former view suggested that service providers who deliver a satisfactory customer experience can lead to the development of consumer trust (Bove and Mitzifiris 2007). However, later studies have reported that trust is an integral factor for ensuring satisfaction in the banking context (Arcand et al. 2017; Omoregie et al. 2019). Given the high levels of perceived risk involved in financial and investment services, a minimum level of trust needs to be established before customers are willing to try out the plethora of financial investment and wealth services. In other words, trust acts as a safety net to help the customer make a financial decision by minimising uncertainty and risk. The insecurity about the long-term horizon for service delivery and the inability to test the service before actual consumption make trust a valuable decision factor for bank customers (Halliburton and Poenaru 2010). Particularly for services such as pension or long-term savings plans which may involve several intangible elements, it may take a minimum of 10 to 20 years before the customer encounters satisfaction/disappointment with the financial product.

In most bank customer–employee interactions, bank customers need to trust their relationship managers before taking up their recommendations on financial products or services. In situations where customers do not trust their relationship managers, they may not heed their financial advice and may not purchase the recommended financial products. Only when they have consumed the products can they evaluate their actual experience with the product or service against their expectations. In this instance, satisfaction or dissatisfaction can only precede when actual consumption is induced by trust. Focusing on high-net-worth banking customers, Boonlertvanich (2019) reported that trust needs to be built for high-wealth customers before they can decide on their preferred or main operating bank. Once a high-wealth customer becomes attached to his or her chosen bank, the formation of customer satisfaction is the key to loyalty behaviours towards the bank. As the study focuses on high-net-worth premium banking customers, trust is considered as an antecedent to satisfaction. Similarly, this relationship has been validated in various consumer studies in the retail banking industry. For example, Pappas et al*.* (2014) reported a positive impact of trust on satisfaction when examining the online shopping experience of retail customers. In recent studies, Rafiq et al. (2020) reported a positive consequence of customer trust on customer satisfaction in listed retail banks in Pakistan, while Suariedewi and Suprapti (2020) found that e-trust displayed a positive and significant effect on e-satisfaction in a study of mobile banking users in Indonesia. In view of the preceding studies, we posit the following hypothesis:

#### H6:

Trust is positively related to satisfaction.

### Trust and customer loyalty

Previous research has reported strong linkages between trust, satisfaction, and customer loyalty (Alnawas and Hemsley-Brown 2018; Boonlertvanich 2019). Prior studies have empirically tested the relationships between trust and loyalty (Chubaka Mushagalusa et al. 2021; Kong et al. 2020; Ha 2020; Lok et al. 2019) and satisfaction and loyalty (Konuk 2018; Prabhakar et al. 2020). Trust plays an imperative role in high contact customer–company relationships, as it creates a bond between the service employee and the customer, making it one of the most influential factors in predicting customer loyalty (Bahadur et al. 2020). In most situations, a customer expects that a company will act in a probable manner, and companies that do not fulfil their customers’ expectations tend to breach their customers’ trust in the relationship (da Rosa Pulga et al. 2019). In an online retailing context, studies have reported a positive relationship between e-trust and e-loyalty, as trust building activities have been found to result in a distinctive level of customer loyalty (Bulut and Karabulut 2018; Khan and Rahman 2016). Focusing on multichannel retailing, Frasquet et al. (2017) found a significant effect of brand trust on both online to offline channel loyalty.

Undeniably, trust plays a paramount role in predicting customer loyalty (Hansen 2014), and the significant effect of trust on loyalty has been confirmed in studies in high contact financial services such as retail banking customers (Kosiba et al. 2018) and commercial banking customers (Ha 2020). The recent case of Wells Fargo Bank, one of the Big Four banks in America, proves the directionality that trust affects loyalty. In 2018, Wells Fargo created millions of fraudulent savings and checking accounts on behalf of its clients without their permission. This account fraud scandal frustrated its customers resulting in a 12% customer defect, as Wells Fargo reported a total of US$1.27 trillion shrinkage in deposits (Egan 2018). To re-establish trust with existing and potential customers, Wells Fargo tried to rebrand and reorganise its company structure. Over time, these efforts paid off and helped the bank turnaround with nearly 59% soar in stock price which not only outperformed the market, but also the broader banking sector. As evident from the case, the Wells Fargo scandal and its turnaround underpins the importance of customer trust, which in turn affect customer loyalty. As the creation of trust between customers and service employees has a positive impact on customer loyalty (Leninkumar 2017), the following hypothesis is advocated:

#### H7:

Trust is positively related to customer loyalty.

### Satisfaction and customer loyalty

Customer satisfaction is regarded as a crucial antecedent of customer loyalty, which is consistent with studies that confirmed the significant effect of customer loyalty on customer satisfaction (Carneiro et al. 2019; Konuk 2018; Prabhakar et al. 2020). For example, Konuk (2018) validated the significant influence of customer satisfaction on repurchase intentions in organic food consumption, while Carneiro et al. (2019) reported that satisfied event attendees are more willing to recommend the event and say positive things about the event, leading to the significant effect of satisfaction on customer loyalty towards the event. In the retailing industry, Audrain-Pontevia and Vanhuele (2016) reported that satisfaction with frontline employee interactions drives customer loyalty for female shoppers, leading to word-of-mouth behaviour and purchase intention. Further, Thakur (2018) confirmed the impact of satisfaction on loyalty in a study of mobile shopping application. As the satisfaction–loyalty linkage has been confirmed by several authors (Leninkumar 2017; Menidjel et al. 2019; Prabhakar et al. 2020), we put forth the following hypothesis:

#### H8:

Satisfaction is positively related to customer loyalty.

### The mediating role of satisfaction

Within the banking literature, the exploration of the mediation effect of satisfaction between trust and loyalty is scare. In a study of hotels, hospitals, and beauty salons in China, Han et al. (2008) reported that customer trust reduced uncertainty and enhanced cumulative satisfaction in relational exchanges. The authors further verified that satisfaction mediated the trust–loyalty relationship. In a study of the retail industry in USA, Taylor et al. (2014) confirmed that satisfaction fully mediated the relationship between trust judgments and future loyalty intentions. Within the mutual fund industry in Taipei, Chiou et al. (2002) validated the partial mediating role of satisfaction in the relationship between trust and loyalty. Further, Mahmoud et al. (2018) found that trust exhibited an indirect significant effect on customer retention via customer satisfaction. With the rise of digital technologies, customer experience in using internet banking, mobile banking, or artificial intelligence (AI) presupposes the mediation effect of satisfaction on the trust–loyalty relationship. Prevalent research suggested that initial trust is a prerequisite to the adoption of financial technologies (i.e., blockchain and cryptocurrency) as customers often face high perceived risk in areas of cyber fraud, cybersecurity, and privacy (Song et al. 2019). Through actual user experience, customer satisfaction increases, which leads to active usage and customer loyalty (Narteh et al. 2022). As trust entails the expectations of customers with the belief that the reliability of banking services can reduce the perceived risks for customers, with enhanced trust in banking products and services, customers who experience the service derive satisfaction with banks, resulting in customer loyalty (Diputra and Yasa 2021). In view of these studies, we formulate the following hypothesis:

#### H9:

Satisfaction mediates the relationship between trust and customer loyalty.

**Fig. 1:**
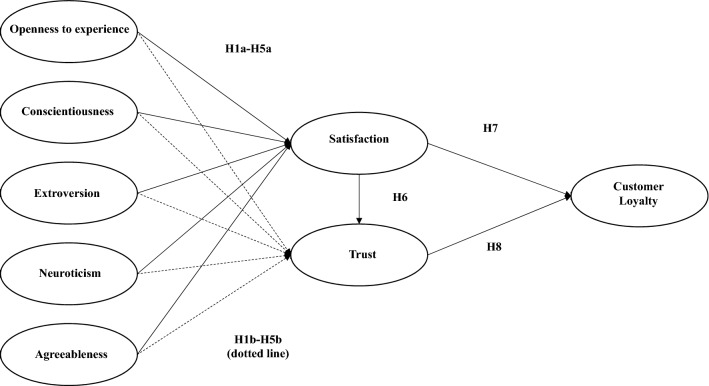
A Model of Customer Personality and its Effect on Trust, Satisfaction and Customer Loyalty

Based on the literature review, we propose a conceptual framework as seen in Fig. 1.

## Research method

### Research setting

The study was conducted in the retail banking industry in Hong Kong, a global financial hub. Hong Kong adopts a three-tier banking system, and banks are divided into three major categories including licensed bank, restricted licensed bank, and deposit taking company. The study targeted clients from the licensed banks. Out of the 159 licensed banks, Hong Kong and Shanghai Banking Corporation and Standard Chartered Bank are the two major note-issuing banks in Hong Kong. In addition to these two banks, respondents from other licensed banks such as Citibank, Bank of China, Bank of East Asia, Hang Seng Bank, among others, were included in the study.

### Sample and data collection

The target of this study consists of high-net-worth premium customers from licensed banks in Hong Kong. According to the survey conducted by Citibank Hong Kong, it was estimated that Hong Kong had about 1.05 million millionaires in 2018 (Yiu 2018). These high-net-worth premium clients represent 14% of banking customers in Hong Kong. A total of 210 online surveys were collected from high-net-worth licensed bank customers through their relationship managers. The respondents in this study consist of premium banking customers with at least HK$1 million assets under management with their bank and were assigned a relationship manager. These customers had interacted with a designated customer contact employee in the preceding year (Eriksson and Hermansson 2017). If the customer had no interaction with any customer contact employee, he or she is classified as a transactional customer and is omitted from this study. To reach the target sample (i.e., high-net-worth banking customers with an existing relationship), purposive sampling was adopted. A short invitation message was sent to the participants via WhatsApp, short message service (SMS), and email, inviting them to participate in an online survey. Due to the restrictions regarding the use of mobile phone numbers legislated by the Data Protection Act in Hong Kong, the invitation message, together with the survey link, was sent out by the local bank relationship managers of the major banks in Hong Kong to their network of premium banking customers.

Table 1 shows the demographic information of all valid responses received. The sample consisted of mainly females (57.10%). Most of the respondents were between 35 and 44 years old (44.30%), 39.5% were between 45 and 54 years old, and 12.9% were above 55 years old. In terms of education level, 47.6% of the respondents held an undergraduate degree while 30% had a postgraduate degree. The respondents were from different occupations such as clerical or administrative (21.40%), manager or executive (21.40%), and professional or consultant (18.60%). Regarding their relationships with their banks, respondents selected Hong Kong and Shanghai Banking Corporation (42.40%) and Standard Chartered Bank (21%) as their major banks. This is not surprising given these banks are the two main note-issuing banks in Hong Kong with a large local client base. The respondents maintained long-term relationships with their relationship managers, with 41.90% of the respondents reported six to 10 years of banking relationship with their relationship managers, while 37.10% mentioned they had over 10 years of banking relationship. After removing 11 invalid samples who did not contact their relationship managers in the preceding year, 210 valid respondents had been in contacted with their relationship manager at least once in the previous year. In terms of frequency, over half of the respondents (54.80%) contacted their relationship managers between three to five times in the past year, while another 39% contacted their relationship managers more than five times, reinforcing the high contact nature of premium banking services.

**Table 1:** Demographic profile of the respondents (*n* = 210)

Variable	Range	Frequency	Percent
Gender	Male	90	42.90
	Female	120	57.10
Age	25–34	7	3.30
	35–44	93	44.30
	45–54	83	39.50
	55 or over	27	12.90
Education	Primary or below	1	0.50
	Secondary	10	4.80
	Diploma/high diploma/associate degree/certificates	36	17.10
	Tertiary/University	100	47.60
	Post-graduate or above	63	30.00
Occupation	Professional/consultant	39	18.60
	Academic	26	12.40
	Technician/operator	24	11.40
	Clerical/administrative	45	21.40
	Manager/executive	45	21.40
	Retired	9	4.30
	Housewife	10	4.80
	Unemployed	1	0.50
	Other	11	5.20
Bank	Standard Chartered Bank	44	21.00
	Hong Kong and Shanghai Banking Corporation	89	42.40
	Hang Seng Bank	28	13.30
	Bank of China	24	11.40
	Other	25	11.90
Relationship duration	1–5 years	44	21.00
	6–10 years	88	41.90
	Over 10 years	78	37.10
Frequency of contact	Less than 3	13	6.20
in past year	3–5 times	115	54.80
	More than 5 times	82	39.00

### Measures

The questionnaire consists of three sections: first, a screening question, second, demographic information, and third, items relating to the constructs. The Big Five, namely openness to experience, conscientiousness, extroversion, neuroticism, and agreeableness, were measured with six items each (i.e., openness to experience, extroversion) and five items each (i.e., conscientiousness, neuroticism, and agreeableness) adopted from Anjam et al. (2013). Trust was assessed using five items taken from Mostafa (2020). Satisfaction was measured with three items adopted from Giovanis et al. (2015), while loyalty was assessed using seven items taken from Givovanis et al. (2015). To operationalise the constructs, seven-point Likert scales, ranging from “1 = strongly disagree” to “7 = strongly agree” were used.

### Pre-test

The survey was pre-tested by professional bankers and academics with expertise in retail banking. Based on their suggestions, minor modifications were made. The revised survey was further pre-tested with four participants, namely two customers from the target group and two relationship managers from the local banks. The results of the pre-test confirmed the face validity and content validity of the instruments. Further, a pilot test was conducted with 52 graduate students. The pilot study established the reliability of the scales, as the values of Cronbach alpha, composite reliability (CR), and average variance extracted (AVE) were satisfactory, achieving construct reliability and validity (Fornell and Larcker 1981; Nunnally 1978).

## Findings

### Reliability and validity

The data for this study were analysed using PLS-SEM 3.3.3 due to its rigorous approach to model assessment as well as the appropriateness of the programme for testing small sample sizes (Hair et al. 2017). Moreover, it is suitable for estimating complex predictive models that assess the strength of the relationships between latent variables with multiple structural paths (Hair et al. 2017). Cronbach alpha and CR were applied to test the reliability of the scales. After the deletion of three items (i.e., conscientiousness item 6, neuroticism item 5, and agreeableness item 5 due to low outer loading of below 0.70), all the remaining 39 items of the outer measurement model had loadings that well exceeded the 0.70 cut-off, demonstrating good convergent validity.

As seen in Table 2, the Cronbach alphas ranged from 0.89 to 0.98 and were above the recommended 0.70 threshold level (Nunnally 1978), while CR ranged from 0.92 to 0.98, which exceeded the 0.70 threshold required for reliability (Bagozzi and Yi 1988). In addition, convergent and discriminant validity were assessed by inspecting the AVE, factor loadings, and CR. All the factor loadings were statistically significant (*p* < 0.001), as all the items demonstrated loadings larger than 0.50 (Hair et al. 2017). As the AVE for all constructs were above 0.5, while the CR were above 0.70, all the items exhibited good internal consistency and high degree of convergence; thus, reliability and validity of measurement scales were supported (Fornell and Larcker 1981).

**Table 2:** Reliability and validity of the constructs

Construct	Item	Standardized factor loading	Cronbach’s alpha	Composite reliability	Average variance extracted
Openness to experience	I enjoy wild flights of fantasy	0.87	0.93	0.95	0.83
	I believe in the importance of art	0.84			
	I experience my emotions intensely	0.81			
	I prefer variety to routine	0.93			
	I love to read challenging materials	0.89			
	I believe that we should be tough on crime	0.89			
Conscientiousness	I complete tasks successfully	0.90	0.92	0.95	0.87
	I like to tidy up	0.85			
	I keep my promises	0.91			
	I work hard	0.90			
	I handle task smoothly	0.89			
Extroversion	I make friends easily	0.93	0.96	0.97	0.88
	I love large parties	0.90			
	I take control of things	0.83			
	I am always busy	0.85			
	I love excitement	0.94			
	I have a lot of fun	0.94			
Neuroticism	I worry about things	0.81	0.89	0.92	0.75
	I get angry easily	0.86			
	I dislike myself	0.88			
	I am afraid to draw attention to myself	0.82			
	I panic easily	0.86			
Agreeableness	I trust others	0.91	0.93	0.96	0.88
	I cheat to get ahead*	0.85			
	I love to help others	0.91			
	I insult people*	0.78			
	I sympathise with the homeless	0.92			
Construct	Item	Standardized factor loading	Cronbach’s Alpha	Composite Reliability	Average Variance Extracted
Trust–competence Trust–integrity Trust–benevolence	I have confidence in my bank’s skills and expertise	0.91	0.97	0.97	0.88
	My bank has the ability to provide for my needs	0.89			
	My bank has been sincere in dealing with me	0.87			
	My bank is completely honest when dealing with me	0.89			
	My bank is always concerned with putting its customers’ interest first	0.90			
Satisfaction	I am satisfied with my experiences with my bank	0.89	0.94	0.96	0.88
	Overall, I am satisfied with my bank	0.82			
	My experience with my bank has exceeded my expectations	0.89			
Loyalty–attitudinal	I do not believe that using other banks is preferable to using my bank	0.90	0.98	0.98	0.89
	I believe that my bank has the best offers at the moment	0.89			
	I prefer the service of my bank to the service of other banks	0.85			
	I have repeatedly found my bank to be better than other banks	0.85			
Loyalty–behavioural	I am a loyal customer of my bank	0.91			
	I have said positive things about my bank to other people	0.92			
	I have recommended my bank to someone who sought my advice	0.92			

*Reversed score items

Discriminant validity was assessed following the Fornell and Larcker (1981) criterion. As seen in Table 3, the square roots of the AVEs of each construct were larger than their respective inter-correlations, confirming the evidence of good discriminant validity. Applying the cross-loadings (Chin 1998) criterion, an item should be highly correlated with its own construct but display low correlations with other constructs.

**Table 3:** Correlations and square root of average variance extracted (diagonal)

	Mean	SD		Open	Cons	Extr	Neur	Agre	Tru	Sat	Loy
Open	5.19	1.08		.91*							
Cons	5.33	1.01		.85*	.93*						
Extr	5.20	1.17		.90*	.80*	.94*					
Neur	2.82	1.01		− .69*	− .59*	− .78*	.87*				
Agre	5.46	1.13		.90*	.85*	.90*	− .72*	.94*			
Tru	5.12	1.18		.85*	.82*	.88*	− .69*	.87*	.94*		
Sat	5.12	1.17		.83*	.78*	.87*	− .68*	.84*	.92*	.94*	
Loy	5.19	1.18		.91*	0.83*	.86*	− .67*	.85*	.93*	.91*	.95*

Open: Openness to experience; Cons: conscientiousness; Extr: extroversion; Neur: neuroticism; Agre: agreeableness; Tru: trust; Sat: satisfaction; Loy: customer loyalty

^*^p < .001

### Common method bias

To reduce common method bias, several procedural remedies suggested by Podsakoff et al. (2003) were adopted. First, the measures for the independent variables (i.e., Big Five dimensions) and the dependent variables (i.e., trust, satisfaction, loyalty) were taken from different sources. Moreover, the respondents were assured of confidentiality and anonymity, as well as any potential risk that might be associated with their participation in the study. In addition, to test for common method bias that may occur due to a cross-sectional survey data obtained from a single source, a common latent construct that was linked to all observed items was included in the measurement model. The results showed that the fit for the measurement model with a common latent construct (SRMR = 0.07) was inferior to the measurement model in this study (SRMR = 0.04). The lack of significance of the method variance verified the absence of common method effects (Podsakoff et al. 2003).

### Structural model

The hypothesised paths in the conceptual framework were estimated using a bootstrapping approach with 5000 resamples in PLS-SEM 3.3.3. The structural model with standardised root mean residual (SRMR) of 0.04, presented a good fit between the conceptual model and the observed data. The predictive ability of the hypothesised model was examined using the following criteria: coefficient of determination (*R*^2^), cross-validated redundancy (Q^2^) and the path coefficients (Hair et al. 2017). As presented in Table 4, the endogenous constructs’ predictive power showed substantial *R*^2^ values of 0.887 (customer loyalty), 0.856 (satisfaction), and 0.813 (trust), which validated the strong predictive power of the model (Hair et al. 2017). A blindfolding approach with an omission distance of eight yielded cross-validated (CV) redundancy was conducted, and the *Q*^2^ values ranged from 0.700 to 0.761 which far exceeded the threshold value of zero, confirming the significance and relevance of the structural model relationships (Hair et al. 2017).

**Table 4:** Hypotheses testing results

Relationship	Path coefficients β	*P* value	Hypothesis testing
H1a openness trust	0.03	0.81	Reject
H1b openness satisfaction	0.03	0.75	Reject
H2a conscientiousness trust	0.24	0.00	Support**
H2b conscientiousness satisfaction	− 0.03	0.71	Reject
H3a extroversion Trust	0.42	0.00	Support***
H3b extroversion satisfaction	0.29	0.01	Support*
H4a neuroticism trust	− 0.04	0.42	Reject
H4b neuroticism satisfaction	0.03	0.48	Reject
H5a agreeableness trust	0.23	0.00	Support**
H5b agreeableness satisfaction	0.02	0.78	Reject
H6 trust satisfaction	0.66	0.00	Support***
H7 trust customer loyalty	0.62	0.00	Support***
H8 satisfaction customer loyalty	0.34	0.00	Support***
*R*^2^ (*Q*^2^) for trust	0.813 (0.700)		
*R*^2^ (*Q*^2^) for satisfaction	0.856 (0.750)		
*R*^2^ (*Q*^2^) for customer loyalty	0.887 (0.761)		

^*^p < .05; **p < .01; ***p < .001

The path coefficient and the *p* values are presented in Table 5. To recap, H1 through H5 addressed the relationships between the five dimensions of customer personality on trust and satisfaction. H1a and H1b, which predicted a positive relationship between openness to experience and trust (*β* = 0.03, *p* = 0.81) and satisfaction (*β* = 0.03, *p* = 0.75), were not supported. H2a, which proposed a positive link between conscientiousness and trust, was supported (*β* = 0.24, *p* < 0.01), but H2b, which proposed a positive correlation conscientiousness and satisfaction, was not supported (*β* = − 0.03, *p* = 0.71). There was a positive link between extroversion and trust (*β* = 0.42, *p* < 0.001) and extroversion and satisfaction (*β* = 0.29, *p* < 0.05), providing support for both H3a and H3b. On the contrary, H4a and H4b, which suggested a positive connection between neuroticism and trust (*β* = − 0.04, *p* = 0.42) and satisfaction (*β* = 0.03, *p* = 0.48) respectively, were both non-significant. H5a which proposed a positive association between agreeableness and trust (*β* = 0.23, *p* < 0.01) was supported, but H5b which suggested a positive link between agreeableness and satisfaction (*β* = 0.02, *p* = 0.78) was not supported. The association between trust and satisfaction was positive and significant (*β* = 0.66, *p* < 0.001), providing support for H6. Finally, H7, which posited a positive link between trust and loyalty (*β* = 0.62, *p* < 0.001) and H8, which proposed a connection between satisfaction and loyalty (*β* = 0.34, *p* < 0.001), were supported.

**Table 5:** Mediation analysis (satisfaction)

Effects	Path (standardised coefficients)	Indirect effect (standard deviation)	Total effect (VAF)	*t*-value (Sig)	Supported yes/no
Direct without mediator	Trustloyalty (0.93)			75.85 (0.00)	
Indirect with mediator (H9)	Trustloyalty (0.63)		0.93 (32.37%)	79.87 (0.00)	Yes, partial mediator
	Trustsatisfaction (0.92)	0.30 (0.08)			
	Satisfactionloyalty (0.33)				

^*^VAF = variance accounted for; VAF > 80% = full mediation, 20% ≤ VAF ≤ 80% = partial mediation, and VAF < 20% = no mediation

### Mediation analysis

As the conceptual model included the mediation effect of satisfaction between trust and loyalty, Baron and Kenny’s (1986) mediation test was applied to assess this mediation relationship. First, the direct effects between both the independent and dependent variables were confirmed with and without the mediator variable (i.e., satisfaction). The path coefficient decreased with the inclusion of the mediator variable. Then, a bootstrapping procedure was undertaken, and the path coefficient and standard error were recorded. A Sobel test (1986) was then performed to assess the indirect effect to determine whether the mediation effect was significant. The strength of the mediation was calculated via the variance accounted for (VAF) method (Hair et al. 2017). The VAF value presented in Table 5 was between 20 and 80%; hence, the partial mediating role of satisfaction was established, providing support for H9.

### Analysis of demographic covariates

Hierarchical multiple regression analysis was performed to investigate the effects of trust and satisfaction on customer loyalty, after controlling for covariates such as age, education level, occupation, gender, bank type, length of relationship, and frequency of contact. Referring to Table 6, in the first block of the hierarchical multiple regression analysis, seven covariates were entered: age, education level, occupation, gender, bank type, length of relationship, and frequency of contact. The model was statistically significant, with *F* (7,210) = 13.78; *p* < 0.001, explaining 32% of the variance in customer loyalty. After the inclusion of trust and satisfaction in Block 2, the total variance increased to 88.80%, with *F* (9,210) = 176.03, *p* < 0.001. The introduction of trust and satisfaction explained an additional 57% of the variance in customer loyalty, after controlling for age, education level, occupation, gender, bank type, length of relationship, and frequency of contact (*R*^2 ^Change = 0.57). The results showed that increased trust (*β* = 3.82, *p* < 0.001) and satisfaction (*β* = 1.89, *p* < 0.001) predicted greater customer loyalty. Among the demographic variables, length of relationship predicted customer loyalty as seen in the significant relationship between them (*β* = 0.52, *p* < 0.05).

**Table 6:** Hierarchical regression model (customer loyalty)

	*R*	*R*^2^	*R*^2^ Change	*B*	SE	*β*	*t*	Sig
Block 1	.59	.32	.32					
Age				− .09	.47	− .01	− .19	.85
Education level				1.19	.51	.16	2.34	.02
Occupation				− .53	.45	− .09	− 1.41	.16
Gender				− .46	.44	− .05	− 1.05	.29
Bank type				.01	.43	.00	.02	.99
Length of relationship				2.42	.53	.34	4.59	.00
Frequency of contact				1.37	.53	.19	2.56	.01
Block 2	.94	.89	.57					
Age				.08	.19	.01	.42	.68
Education level				.13	.21	.02	.63	.53
Occupation				.05	.18	.00	.27	.79
Gender				− .14	.18	− .02	− .79	.43
Bank type				.84	.18	.01	.48	.63
Length of relationship				.52	.23	.07	2.28	.02
Frequency of contact				− .07	.22	− .01	− .30	.77
Trust				3.82	.37	.62	10.40	.00
Satisfaction				1.89	.37	.31	5.07	.00

*R*^2^ = Amount of variance explained by independent variables

*R*^2^ Change = additional variance in dependent variables

*B* = Unstandardized coefficient

SE = Standard error

*β* = Standardized coefficient

*t* = Estimated coefficient (B) divided by its own standard error

## Discussion and implications

The findings demonstrated the influence of the Big Five personality (McCrae and Costa 1987) on trust, satisfaction, and customer loyalty, with trust and satisfaction displaying a significant effect on customer loyalty. The results reinforced the importance of customer personality in interpersonal interactions in a premium banking services context (Castillo 2017; Mishra and Vaithianathan 2015; Mohammad 2014).

First, the findings indicated that openness to experience had no impact on both trust (H1a) and satisfaction (H1b) which contradict existing literature (Castillo 2017; Karbasi et al. 2014; Malik et al. 2018). As openness to experience describes the level which an individual is open to new ideas and change (Goldberg 1981), this might not be relevant in high risk, premium investment and wealth services, as most high-net-worth clients might not be willing to try out new investment products without careful evaluation and extended decision making. This can be explained by the law of diminishing marginal utility of wealth which indicated that the higher the wealth level, the more the risk aversion displayed by an individual, and consequently, the smaller the increase in satisfaction and happiness (Marshall 1890). Thus, the wealth level of high-net-wealth customers may be a better indicator compared to openness to experience in explaining their financial decisions. This could be the reason for the lack of relationships between openness to experience, trust, and satisfaction.

Second, both conscientiousness (H2a) and agreeableness (H5a) show positive relationships with trust which are consistent with previous studies (Siddiqui 2016). As conscientiousness assessed a person’s level of organisation and perseverance, customers with high levels of conscientiousness often display a high preference for organisation and reliability (Mishra and Vaithianathan 2015). Given that retail banking services commonly involve processes consisting of precious data, exhaustive documentation, and comprehensive verifications, these detail-oriented services are likely to gratify conscientious customers in their quest for data, logical reasoning, and determination, thereby resulting in higher levels of trust (Mishra and Vaithianathan 2015). The level of trust tends to be immense for wealthy customers who demonstrate higher levels of conscientiousness as they possess specific skills that facilitate status achievement (Leckelt et al. 2019). In addition, customers with high levels of agreeableness are extremely affectionate, cooperative, and display prosocial behaviours, and their empathy, care, and concern for others often result in greater notions of trust during customer-employee interactions (Mishra and Vaithianathan 2015). Moreover, McCrae and Costaa (1987) suggested that individuals that are high on agreeableness tend to trust and believe in the best of others, and rarely display suspicious hidden intents. Thus, agreeableness is often accompanied by high levels of trust in relationship managers.

Third, the results verified the predictive power of extroversion on both trust (H3a) and satisfaction (H3b). This is not surprising, as extroverts are outgoing, sociable, active, chatty and prefer to engage with others, leading to perceptions of trust and satisfaction. In addition, an affirmative relationship between extroversion, trust, and satisfaction had been reported in previous studies (Castillo 2017; Mishra and Vaithianathan 2015), where high levels of extroversion were evident in affluent banking service encounters that require a great deal of social exchanges between the customer and the employee, resulting in the formation of social bonds and friendships which contributed to consumer trust and satisfaction. Specifically, trust and satisfaction may be magnified as high-net-wealth individuals are typically characterised by active personality or higher level of extroversion as they strive for power, status and “getting ahead” (Leckelt et al. 2019). Moreover, prestige-based premium banking services often portray the image of high financial prominence which is well aligned to the status seeking personality (i.e., extroversion) of millionaires.

Fourth, the findings did not establish any relationships between neuroticism and trust (H4a) nor satisfaction (H4b). Examining the research context, the relationships between neuroticism and trust/satisfaction were confirmed in non-banking contexts (Castillo 2017; Durukan and Bozaci 2011) but not in banking contexts (Karbasi et al. 2014; Mishra and Vaithianathan 2015). As banking services might include investment advisory services, with unstable and highly volatile investment performance outcomes, the nature of the service might easily trigger the emotions of neurotic individuals who tend to be more sensitive and easily affected by emotions which might impact on their levels of trust and satisfaction. Further, some studies have reported the negative influence of emotions on trust (Lee and Selart 2012). As neurotic clients are marked by fear, aggression, pessimism, depression, and anxious behaviour (McCrae and Costa 1987), they tend to place more importance on the negative aspects of information and avoid risky investments which in turn, lead to dissatisfaction and distrust when they experience loss in the actual portfolio returns (Husnain et al. 2019).

Fifth, in terms of outcomes, trust displayed a significant influence on satisfaction (H6). This is supported by several studies which found trust and satisfaction as important determinants of customer loyalty (Agnihotri et al. 2019; Omoregie et al. 2019; Yildiz 2017). In this study, trust emerged as a stronger predictor of customer loyalty (H7) compared to satisfaction (H8), which partially mediated the relationship between trust and loyalty (H9). In the premium banking industry, trust is forged when consumers can rely on a certain level of acumen among financial service providers. Further, the increased government regulations in the finance industry have led to a rise in professional practices by banks, including relationship management strategies and trust-building efforts, resulting in enhanced customer satisfaction. When customer expectations are fulfilled and exceeded, satisfying experiences are created, leading to customer loyalty to the banks (Hansen 2014; Menidjel et al. 2019).

Finally, in terms of the effects of demographic characteristics, the results showed that relationship length had a positive influence on customer loyalty, which is consistent with previous studies (Barnes 1997; Bove and Johnson 2009). Customers with positive experiences over time tend to forgive more and are less likely to defect from the banking relationship. As a vital relationship variable, relationship length directly affects the profitability of banks, since the in-depth knowledge of existing customers helps the banks identify customer needs and increase cross selling opportunities (Fredriksson and Moro 2014). Thus, it is integral that banks seek to develop and maintain long-term relationships with their key customers.

### Theoretical implications

This study clarifies the inconsistent results of previous studies (Bove and Mitzifiris 2007; Jani and Ha 2014), and confirms the significant impact of conscientiousness, extroversion, and agreeableness on trust, as well as the effect of extroversion on satisfaction. In doing so, this study addresses the void by elucidating the role of customer personality in CRM in a high-net-worth banking services context. Unsurprisingly, the personalities of high-net-worth individuals are different from the general population (Leckelt et al. 2019), yet insights into high-net worth individuals are lacking despite its emerging importance as a possible target segment for banks (Schroder et al. 2020). This study contributes to the existing literature regarding consumer behaviours of high-net-worth individuals or groups by clarifying the personality dimensions that affect trust and satisfaction. In addition, the study contributes to the field of services research by clarifying the predictive power of the Big Five personality traits (McCrae and Costa 1987) on the trust–satisfaction–loyalty link and pinpointing the important dimensions of customer personality that affect feelings of customer trust, satisfaction, and loyalty. Insights into the personality–trust–loyalty and personality–satisfaction–loyalty links can act as a starting point for academics who are interested in understanding the role of customer personality in consumer behaviour and decision making.

In addition, the validation of the mediation effect of satisfaction between trust and loyalty further shed light on the importance of trust in predicting customer loyalty in the banking context, both in its direct effect on loyalty, as well as its indirect effect via satisfaction. As there is a lack of banking studies that investigate this effect, the mediating analysis adds value to academics who are keen in studying the customer relationship formation process and its related relationship marketing variables. One should note that providing financial services is different from providing standardised services like retailing, hospitality, and tourism services. For example, customers seeking hospitality services normally expect the same level of service from one period to another, while customers of financial services are harder to please as the performance of investment products tend to fluctuate over time (Rajaobelina and Bergeron 2009). Thus, the building of trust becomes of utmost importance in an affluent banking services context, where banks need to proactively tailor their products and services in anticipation of their customers’ increasing expectations and evolving needs.

### Practical implications

The findings render practical insights for retail bank managers who are involved in the management of customer relationships. Given the significant associations between trust and customer loyalty, managers should prioritise their relationship management efforts in trust-building activities to bring about long-term customer relationships. To increase consumer trust, retail banks can invest in extensive financial and wealth management training to equip their financial advisors with the right expertise to provide sound financial advice and guidance that is genuinely good for their customers and strive to constantly put customers’ interests at heart. This can create value for their affluent customers especially during the COVID-19 pandemic, where customer-contact employee professionalism and customer relationships are essential to elevate the levels of trust of their affluent banking clients (Lin et al. 2021).

In view of the significant relationships between extroversion on trust and satisfaction, banks should use different types of marketing communications to engage the extroverts and introverts. For example, employees can communicate via the telephone or face-to-face with extroverts, while email exchanges or messages via the online banking website or app can be used for communicating with introverts. To enhance service delivery, banks should provide different servicescapes (i.e., physical environment elements) for extroverted and introverted customers. Branches offering a comfortable face-to-face banking experience which encourages social interactions could fulfil the needs of extroverts while virtual banks with full banking facilities could engage introverts.

This study validated the effect of trust on both satisfaction and customer loyalty. Based on the importance of trust, managers should direct every effort to trust-building via professionalism which can promote long-lasting relationship in high contact, relational services (Balaji Rao and Rao 2019). Managers should also devote more resources to customer service training to enhance personnel quality so that employees can handle customers in a professional and competent manner, thus enhancing customer relationships and increasing trust and satisfaction (Al-Salim 2018; Lucia-Palacios et al. 2020). In addition, banks can increase loyalty by implementing personality-targeted marketing strategies as some personalities such as extraverts are known to better respond to personality-matched marketing and advertising (Moss 2017).

In the era of Industry 4.0, banks can consider investing in AI and/or FinTech to help relationship managers in their actual service delivery (Jaiwant 2022). With the aid of AI, bank employees can easily recognise the various customer personalities to better formulate communication strategies to meet customers’ needs and service requirements. Depending on the personality profiles of customers identified through the help of sophisticated AI tools (Singh et al. 2019), employees can adjust their contact methods, message content, and frequency of conversations and dialogues to suit their affluent clients for better service performance and relationship success. With the ease of access to behavioural and psychological segmentation and targeting tools and methods, data collected from technologically connected internet-of-things (IoT) such as mobile apps, search engines, and social media platforms can be further integrated for personality-targeted marketing. In particular, data collected from search queries, social media, purchasing patterns, and online browsing history can allow accurate targeting of customers based on their personality traits, leading to customer satisfaction and loyalty (Itani et al. 2020).

### Limitations and future research

Given the time and budget constraint, the study collected only 210 valid samples. Further studies can collect larger samples across premium banking services in different countries for better generalisation. The current study adopted a cross-sectional survey with a non-probability sampling method, thus future studies could consider longitudinal studies that can extend the scope of this study. This research focused solely on relational customers who have an existing relationship with their relationship manager. Future studies could examine whether transactional customers display similar relational attributes (Eriksson and Hermansson 2017). To further generalise the findings, future studies could delve into the role of customer personality as well as additional dimensions such as risk aversion, commitment, empowerment, and engagement from various perspectives (i.e., contact employee and customer) in different industries or countries (i.e., developed to developing) with different cultures and sub-cultures (i.e., age, gender, social class), using a mix of quantitative and qualitative research methods. In light of the recent digital disruptions, future research can focus on how technology (i.e., AI, machine learning, cloud computing, IoT) can be harnessed to facilitate the delivery of high-quality, premium banking services.

## References

[CR1] Al-Hawari MAA (2014). Does customer sociability matter? Differences in e-quality, e-satisfaction, and e-loyalty between introvert and extravert online banking users. Journal of Services Marketing.

[CR2] Al-Salim MIA (2018). A closer look at the relationship of entry-level bank employees’ leadership attributes and customer satisfaction. Journal of Financial Services Marketing.

[CR3] Abrar M, Biag SA, Shabbir R, Hussain I (2019). Role of CRM practices in bank’s performance: Moderating role of market turbulence. Journal of Managerial Sciences.

[CR4] Agnihotri R, Yang Z, Briggs E (2019). Salesperson time perspectives and customer willingness to pay more: Roles of intraorganisational employee navigation, customer satisfaction, and firm innovation climate. Journal of Personal Selling and Sales Management.

[CR6] Alnawas I, Hemsley-Brown J (2018). The differential effect of cognitive and emotional elements of experience quality on the customer-service provider's relationship. International Journal of Retail and Distribution Management.

[CR7] Amin M (2016). Internet banking service quality and its implication on e-customer satisfaction and e-customer loyalty. International Journal of Bank Marketing.

[CR8] Amin M, Isa Z, Fontaine R (2013). Islamic banks: Contrasting the drivers of customer satisfaction on image, trust, and loyalty of Muslim and non-Muslim customers in Malaysia. International Journal of Bank Marketing.

[CR9] Anjam M, Siddiqui K, Khan SS (2013). Selection of a survey research instrument: Impediments of personality inventory in non-English speaking countries like Pakistan. European Journal of Business Management.

[CR10] Arcand M, PromTep S, Brun I, Rajaobelina L (2017). Mobile banking service quality and customer relationships. International Journal of Bank Marketing.

[CR11] Audrain-Pontevia A, Vanhuele M (2016). Where do customer loyalties really lie, and why? Gender differences in store loyalty. International Journal of Retail and Distribution Management.

[CR12] Bagozzi RP, Yi Y (1988). On the evaluation of structural equation models. Journal of Academy of Marketing Science.

[CR13] Bahadur W, Khan AN, Ali A, Usman M (2020). Investigating the effect of employee empathy on service loyalty: The mediating role of trust in and satisfaction with a service employee. Journal of Relationship Marketing.

[CR14] Balaji Rao DG, Rao BR (2019). Professionalism and ethics in financial advising. Journal of International Management Studies.

[CR15] Barnes JG (1997). Closeness, strength, and satisfaction: Examining the nature of relationships between providers of financial services and their retail customers. Psychology Marketing.

[CR16] Baron RM, Kenny DA (1986). The moderator–mediator variable distinction in social psychological research: Conceptual, strategic, and statistical considerations. Journal of Personality and Social Psychology.

[CR17] Bawack RE, Wamba SF, Carillo KDA (2021). Exploring the role of personality, trust, and privacy in customer experience performance during voice shopping: Evidence from SEM and fuzzy set qualitative comparative analysis. International Journal of Information Management.

[CR18] Block J (1995). A contrarian view of the five-factor approach to personality description. Psychological Bulletin.

[CR19] Boonlertvanich K (2019). Service quality, satisfaction, trust, and loyalty: The moderating role of main-bank and wealth status. International Journal of Bank Marketing.

[CR20] Bove L, Mitzifiris B (2007). Personality traits and the process of store loyalty in a transactional prone context. Journal of Services Marketing.

[CR21] Bove LL, Johnson LW (2009). Does “true” personal or service loyalty last? A longitudinal study. Journal Service Marketing.

[CR22] Brun I, Rajaobelina L, Ricard L (2014). Online relationship quality, scale development and initial testing. International Journal of Bank Marketing.

[CR23] Brun I, Rajaobelina L, Ricard L (2016). Online relationship quality: Testing an Iintegrative and comprehensive model in the banking industry. Journal of Relationship Marketing.

[CR24] Bulut ZA, Karabulut AN (2018). Examining the role of two aspects of eWOM in online repurchase intention: An integrated trust–loyalty perspective. Journal of Consumer Behavior.

[CR25] Caliskan A (2019). Applying the right relationship marketing strategy through big five personality traits. Journal of Relationship Marketing.

[CR26] Carneiro MJ, Eusébio C, Caldeira A, Santos AC (2019). The influence of eventscape on emotions, satisfaction and loyalty: The case of re-enactment events. International Journal of Hospitality Management.

[CR27] Castillo J (2017). The relationship between big five personality traits, customer empowerment and customer satisfaction in the retail industry. Journal of Business and Retail Management Research (JBRMR).

[CR28] Chai JCY, Malhotra NK, Alpert F (2015). A two-dimensional model of trust-value-loyalty in service relationships. Journal of Retailing and Consumer Services.

[CR31] Chin, W.W. (1998) The partial least squares approach to structural equation modelling. In *Modern methods for business research*. G.A. Marcoulides ed. M. L. Erlbaum, 295–358*.*

[CR32] Chiou JS, Droge C, Hanvanich S (2002). Does customer knowledge affect how loyalty is formed?. Journal of Service Research.

[CR33] Choi L, Hwang J (2019). The role of prosocial and proactive personality in customer citizenship behaviors. Journal of Consumer Marketing.

[CR34] Chubaka Mushagalusa N, Balemba Kanyurhi E, Akonkwa Bugandwa Mungu D (2021). Measuring price fairness and its impact on consumers’ trust and switching intentions in microfinance institutions. Journal of Financial Services Marketing.

[CR35] Crawford EC, Jackson J, Pritchard A (2017). A more personalized satisfaction model: Including the BFI-44 in the American Customer Satisfaction Model. Journal of Consumer Satisfaction, Dissatisfaction and Complaining Behavior.

[CR36] da Rosa Pulga AAR, Basso K, Viacava KR, Pacheco NA, Ladeira WJ, Dalla Corte VF (2019). The link between social interactions and trust recovery in customer–business relationships. Journal of Consumer Behavior.

[CR37] Dick AS, Basu K (1994). Customer loyalty: Toward an integrated conceptual framework. Journal of the Academy of Marketing Science.

[CR38] Diputra IGAW, Yasa NN (2021). The influence of product quality, brand image, brand trust on customer satisfaction and loyalty. American International Journal of Business Management (AIJBM).

[CR39] Durukan, T., and I. Bozaci. 2011. The role of individual characteristics on customer loyalty. *International Journal of Business and Social Science* 2(23).

[CR40] Egan, M. 2018. Wells Fargo customers are fed up. They cound yank billions of dollars in deposits Retrieved from https://edition.cnn.com/2018/10/10/business/wells-fargo-bank-customers-scandal/index.html

[CR41] Ennew C, Waite N, Waite R (2018). Financial services marketing: An international guide to principles and practice.

[CR42] Ennew C, Sekhon H (2007). Measuring trust in financial services; the Trust Index. Consumer Policy Review.

[CR43] Eriksson K, Hermansson C (2017). Do consumers subjectively perceive relationships in objectively defined relational, interimistic, and transactional exchange in financial services?. International Journal of Bank Marketing.

[CR44] Evans AM, Revelle W (2008). Survey and behavioral measurements of interpersonal trust. Journal of Research in Personality.

[CR45] Fornell C, Larcker DF (1981). Evaluation structural equality models with unobserved variables and measurement error. Journal of Marketing Research.

[CR46] Frasquet M, Mollá Descals A, Ruiz-Molina M (2017). Understanding loyalty in multichannel retailing: The role of brand trust and brand attachment. International Journal of Retail and Distribution Management.

[CR47] Fredriksson A, Moro A (2014). Bank–SMEs relationships and banks’ risk-adjusted profitability. Journal of Banking Finance.

[CR48] Freitag M, Bauer PC (2016). Personality traits and the propensity to trust friends and strangers. The Social Science Journal.

[CR50] Giovanis A, Athanasopoulou P, Tsoukatos E (2015). The role of service fairness in the service quality-relationship quality-customer loyalty chain: An empirical study. Journal of Service Theory and Practice.

[CR51] Goldberg, L.R. 1981. Language and individual differences: the search for universals. In *Review of personality and social psychology.* Personality lexicons in Wheeler ed*.* 1:141–165. Beverly Hills, CA: Sage.

[CR175] Gokmenoglu, K. K., and A. Amir. 2021. The impact of perceived fairness and trustworthiness on customer trust within the banking sector. *Journal of Relationship Marketing* 20 (3): 241–260.

[CR52] Ha V (2020). The effects of attitude, trust and switching cost on loyalty in commercial banks in Ho Minh City. Accounting.

[CR53] Han E, Kim KK, Lee AR (2019). Contributors to exchange structures and their effects on community solidarity in online communities. Internet Research.

[CR54] Hair JF, Hult GTM, Ringle CM, Sarstedt M (2017). A primer on partial least squares structural equation modeling (PLS-SEM).

[CR55] Halliburton C, Poenaru A (2010). The role of trust in consumer relationships. ESCP Europe Business School.

[CR56] Han X, Kwortnik RJ, Wang C (2008). Service loyalty: An integrative model and examination across service contexts. Journal of Service Research.

[CR57] Hansen H, Sand J (2008). Antecedents to customer satisfaction with financial services: The moderating effects of the Need to Evaluate. Journal of Financial Services Marketing.

[CR58] Hansen T (2014). The role of trust in financial customer–seller relationships before and after the financial crisis. Journal of Consumer Behavior.

[CR59] Hansemark OC, Albinsson M (2004). Customer satisfaction and retention: The experiences of individual employees. Managing Service Quality: An International Journal.

[CR60] Harrigan P, Evers U, Miles MP, Daly T (2018). Customer engagement and the relationship between involvement, engagement, self-brand connection and brand usage intent. Journal of Business Research.

[CR61] Herman LE, Sulhaini S, Farida N (2020). Electronic customer relationship management and company performance: Exploring the product innovativeness development. Journal of Relationship Marketing.

[CR62] Hiraishi K, Yamagata S, Shikishima C, Ando J (2008). Maintenance of genetic variation in personality through control of mental mechanisms: A test of trust, extraversion, and agreeableness. Evolution and Human Behavior.

[CR63] Homans GC (1958). Social behavior as exchange. American Journal of Sociology.

[CR64] Hsu CL, Chen MC, Kumar V (2018). How social shopping retains customers? Capturing the essence of website quality and relationship quality. Total Quality Management and Business Excellence.

[CR65] Husnain B, Shah SZA, Fatima T (2019). Effect of neuroticism, conscientiousness on investment decisions. Mediation analysis of financial self-efficacy. City University Research Journal.

[CR66] Im J, Qu H, Beck JA (2021). Antecedents and the underlying mechanism of customer intention of co-creating a dining experience. International Journal of Hospitality Management.

[CR67] Itani OS, El Haddad R, Kalra A (2020). Exploring the role of extrovert-introvert customers’ personality prototype as a driver of customer engagement: does relationship duration matter?. Journal of Retailing and Consumer Services.

[CR68] Jaiwant, S. V. (2022). Artificial intelligence and personalized banking. In *Handbook of**research on innovative management using AI in industry 5.0*. 74–87. IGI Global.

[CR69] Jani D, Han H (2014). Personality, satisfaction, image, ambience, and loyalty: Testing their relationships in the hotel industry. International Journal of Hospitality Management.

[CR70] Jeon HG, Kim C, Lee J, Lee KC (2021). Understanding E-commerce consumers’ repeat purchase intention: The role of trust transfer and the moderating effect of neuroticism. Frontiers in Psychology.

[CR71] Ji C, Prentice C (2021). Linking transaction-specific satisfaction and customer loyalty: The case of casino resorts. Journal of Retailing and Consumer Services.

[CR72] John OP, Srivastava S, Pervin L, John OP (1999). The big-five trait taxonomy: History, measurement, and theoretical perspectives. Handbook of personality theory and research.

[CR73] Johnson D, Grayson K (2005). Cognitive and affective trust in service relationships. Journal of Business Research.

[CR74] Jung JH, Yoo J (2019). The effects of deviant customer-oriented behaviors on service friendship: The moderating role of co-production. Journal of Retailing and Consumer Services.

[CR75] Karbasi A, Navid BJ, Hashemi SR (2014). The influence of personality traits of customers on their loyalty: A case study of private banks of Kermanshah. International Research Journal of Applied and Basic Sciences.

[CR76] Kaur SJ, Ali L, Hassan MK (2021). Adoption of digital banking channels in an emerging economy: Exploring the role of in-branch efforts. Journal of Financial Services Marketing.

[CR77] Khan I, Rahman Z (2016). E-tail brand experience’s influence on e-brand trust and e-brand loyalty. International Journal of Retail and Distribution Management.

[CR78] Kim SH, Kim M, Holland S (2018). How customer personality traits influence brand loyalty in the coffee shop industry: The moderating role of business types. International Journal of Hospitality and Tourism Administration.

[CR79] Kim H, Qu H (2020). The mediating roles of gratitude and obligation to link employees’ social exchange relationships and prosocial behaviour. International Journal of Contemporary Hospitality Management.

[CR81] Kong Y, Wang Y, Hajli S, Featherman M (2020). In sharing economy we trust: Examining the effect of social and technical enablers on millennials’ trust in sharing commerce. Computers in Human Behavior.

[CR82] Konuk FA (2018). Price fairness, satisfaction, and trust as antecedents of purchase intentions towards organic food. Journal of Consumer Behavior.

[CR83] Kosiba J, Boateng H, Okoe Amartey A, Boakye R, Hinson R (2018). Examining customer engagement and brand loyalty in retail banking. International Journal of Retail and Distribution Management.

[CR84] Kreuzer M, Gollwitzer M (2021). Neuroticism and satisfaction in romantic relationships: A systematic investigation of intra-and interpersonal processes with a longitudinal approach. European Journal of Personality.

[CR85] Lambe CJ, Wittman CM, Spekman RE (2001). Social exchange theory and research on business-to-business relational exchange. Journal of Business to Business Marketing.

[CR86] Lainamngern S, Sawmong S (2019). How customer relationship management, perceived risk, perceived service quality, and passenger trust affect a full-service airline’s passenger satisfaction. Journal of Business and Retail Management Research.

[CR87] Leckelt M, Richter D, Schröder C, Küfner AC, Grabka MM, Back MD (2019). The rich are different: Unravelling the perceived and self-reported personality profiles of high-net-worth individuals. British Journal of Psychology.

[CR88] Lee JJ, Capella ML, Taylor CR, Luo M, Gabler CB (2014). The financial impact of loyalty programs in the hotel industry: A social exchange theory perspective. Journal of Business Research.

[CR89] Lee WS, Selart M, Hauppage KOM, Gonzalez NP (2012). The impact of emotions on trust decisions. Handbook on psychology of decision-making.

[CR90] Leninkumar V (2017). The relationship between customer satisfaction and customer trust on customer loyalty. International Journal of Academic Research in Business and Social Sciences.

[CR91] Lewis JD, Weigert A (1985). Trust as a social reality. Social Forces.

[CR92] Lin AJ, Chang HY, Huang SW (2021). Criteria affecting Taiwan wealth management banks in serving high-net-worth individuals during COVID-19: A DEMATEL approach. Journal of Financial Services Marketing.

[CR93] Lin YL (2010). The relationship of consumer personality trait, brand personality and brand loyalty: An empirical study of toys and video games buyers. Journal of Product and Brand Management.

[CR94] Lin Y, Worthley R (2012). Serviscape moderation on personality traits, emotions, satisfaction, and behaviors. International Journal of Hospitality Management.

[CR95] Liyanaarachchi G, Deshpande S, Weaven S (2021). Online banking and privacy: Redesigning sales strategy through social exchange. International Journal of Bank Marketing.

[CR96] Lok S, Vui C, Chuen S, Wei L (2019). Modelling perceived value, trust, satisfaction and consumer commitment: An analysis of fast-moving consumer goods in Malaysia. Global Business and Management Research.

[CR97] Lucia-Palacios L, Pérez-López R, Polo-Redondo Y (2020). How situational circumstances modify the effects of frontline employees’ competences on customer satisfaction with the store. Journal of Retailing and Consumer Services.

[CR99] Malik AM, Hairuddin H, Shuib N (2018). Openness to experience—A moderator between social commerce success factors and customer satisfaction relationship: Facebook brand page platform. Management and Accounting Review.

[CR100] Mahmoud MA, Hinson RE, Adika MK (2018). The effect of trust, commitment, and conflict handling on customer retention: The mediating role of customer satisfaction. Journal of Relationship Marketing.

[CR101] Malvika, C. 2022. Mortgage delinquency during COVID-19: Do financial literacy and personality traits matter? *International Journal of Bank Marketing* Vol. ahead-of-print No. ahead-of-print.

[CR102] Mann BJS, Rawat J (2016). The role of consumer personality trait and brand personality trait in creating customer experience. IUP Journal of Brand Management.

[CR103] Marshall A (1890). Principles of economics.

[CR105] McAllister DJ (1995). Affect-and cognition-based trust as foundations for interpersonal cooperation in organizations. Academy of Management Journal.

[CR106] McCrae RR, Costa PT (1987). Validation of the five-factor model of personality across instruments and observers. Journal of Personality and Social Psychology.

[CR108] Mehl MR, Gosling SD, Pennebaker JW (2006). Personality in its natural habitat: Manifestations and implicit folk theories of personality in daily life. Journal of Personality and Social Psychology.

[CR109] Menidjel C, Bilgihan A, Benhabib A (2021). Exploring the impact of personality traits on perceived relationship investment, relationship quality, and loyalty in the retail industry. The International Review of Retail, Distribution and Consumer Research.

[CR110] Menidjel C, Benhabib A, Bilgihan A, Madanoglu M (2019). Assessing the role of product category involvement and relationship proneness in the satisfaction–loyalty link in retailing. International Journal of Retail and Distribution Management.

[CR111] Mishra V, Vaithianathan S (2015). Customer personality and relationship satisfaction: Empirical evidence from Indian banking sector. International Journal of Bank Marketing.

[CR112] Moghavvemi S, Woosnam KM, Hamzah A, Hassani A (2021). Considering residents’ personality and community factors in explaining satisfaction with tourism and support for tourism development. Tourism Planning and Development.

[CR113] Mohammad A (2014). Does customer sociability matter? Differences in e-quality, e-satisfaction, and e-loyalty between introvert and extrovert online banking users. Journal of Services Marketing.

[CR114] Mohammad A (2015). How the personality of retail bank customers interferes with the relationship between service quality and loyalty. The International Journal of Bank Marketing.

[CR115] Moorman C, Deshpande R, Zaltman G (1993). Factors affecting trust in market research relationship. The Journal of Marketing.

[CR116] Mostafa RB (2020). Mobile banking service quality: A new avenue for customer value co-creation. International Journal of Bank Marketing.

[CR117] Morgan RM, Hunt SD (1994). The commitment-trust theory of relationship marketing. Journal of Marketing.

[CR118] Moss G (2017). Personality, design and marketing: Matching design to customer personal preferences.

[CR119] Mukerjee K (2018). The impact of brand experience, service quality and perceived value on word of mouth of retail bank customers: Investigating the mediating effect of loyalty. Journal of Financial Services Marketing.

[CR120] Narteh B, Braimah M (2019). Corporate reputation and retail bank selection: The moderating role of brand image. International Journal of Retail and Distribution Management.

[CR121] Narteh B, Yeboah-Asiamah E, Mackin EA (2022). Analysis of young banked and unbanked customers' usage, satisfaction, trust and loyalty for mobile money services in Ghana. International Journal of Business and Systems Research.

[CR122] Nora L (2019). Trust, commitment, and customer knowledge: Clarifying relational commitments and linking them to repurchasing intentions. Management Decision.

[CR123] Nunnally, J.C. 1978. Assessment of reliability. In* Psychometric theory*, 2nd edn. New York: McGraw-Hill.

[CR124] Olavarría-Jaraba A, Cambra-Fierro JJ, Centeno E, Vázquez-Carrasco R (2018). Analyzing relationship quality and its contribution to consumer relationship proneness. Services Business.

[CR125] Oliver R (1993). A conceptual model of service quality and service satisfaction: Compatible goals, different concepts. Advances in Services Marketing and Management.

[CR126] Oliver R (1997). Satisfaction: A behavioral perspective on the consumer.

[CR127] Omoregie OK, Addae JA, Coffie S, Ampong GOA, Ofori KS (2019). Factors influencing consumer loyalty: Evidence from the Ghanaian retail banking industry. International Journal of Bank Marketing.

[CR128] Patitsa CD, Sahinidis AG, Tsaknis PA, Giannakouli V (2021). Big five personality traits and students satisfaction with synchronous online academic learning (SOAL). Corporate Business Strategy Review.

[CR129] Podsakoff PM, MacKenzie SB, Lee JY, Podsakoff NP (2003). Common method biases in behavioral research: A critical review of the literature and recommended remedies. Journal of Applied Psychology.

[CR130] Prabhakar G, Yeong SN, Knox D (2020). Customer satisfaction and loyalty in Malaysian resort hotels: The role of empathy, reliability and tangible dimensions of service quality. International Journal of Services and Operations Management.

[CR131] Purani K, Kumar D, Sahadev S (2019). E-Loyalty among millennials: Personal characteristics and social influences. Journal of Retailing and Consumer Services.

[CR132] Putra IWJA, Putri DP (2019). The mediating role of relationship marketing between service quality and customer loyalty. Journal of Relationship Marketing.

[CR133] Rafiq MZ, Jun JC, Ali R, Majeed MK, Mohsin M (2020). Impact of corporate image, switching cost and customer trust on customer satisfaction: Evidence from listed banking sector. SMART Journal of Business Management Studies.

[CR135] Rajaobelina L, Bergeron J (2009). Antecedents and consequences of buyer-seller relationship quality in the financial services industry. The International Journal of Bank Marketing.

[CR136] Reichheld FF (2003). The one number you need to grow. Harvard Business Review.

[CR137] Roy S, Sethuraman R, Saran R (2016). The effect of demographic and personality characteristics on fashion shopping proneness. International Journal of Retail and Distribution Management.

[CR138] Schröder C, Bartels C, Grabka MM, König J, Kroh M, Siegers R (2020). A novel sampling strategy for surveying high-net-worth individuals—A pretest application using the socio-economic panel. Review of Income and Wealth.

[CR139] Siddiqui KA (2016). Personality influences customer loyalty. Science International (lahore).

[CR140] Singh J, Flaherty K, Sohi RS, Deeter-Schmelz D, Habel J, Le Meunier-FitzHugh K, Onyemah V (2019). Sales profession and professionals in the age of digitization and artificial intelligence technologies: Concepts, priorities, and questions. Journal of Personal Selling and Sales Management.

[CR141] Smith, T.A. 2020. The role of customer personality in satisfaction, attitude-to-brand and loyalty in mobile services. *Spanish Journal of Marketing-ESIC.*

[CR142] Singh D, Bajpai N, Kulshreshtha K (2020). Brand experience-brand love relationship for Indian hypermarket brands: The moderating role of customer personality traits. Journal of Relationship Marketing.

[CR143] Sobel ME (1986). Some new results on indirect effects and their standard errors in covariance structure. Sociology Methodology.

[CR144] Solomon MR (2018). Consumer behavior: Buying, having, and being.

[CR145] Song H, Wang J, Han H (2019). Effect of image, satisfaction, trust, love, and respect on loyalty formation for name-brand coffee shops. International Journal of Hospitality Management.

[CR146] Spake D, Megehee C (2010). Consumer sociability and service provider expertise influence on service relationship success. Journal of Services Marketing.

[CR147] Strandberg C, Wahlberg O, Öhman P (2015). Effects of commitment on intentional loyalty at the person-to-person and person-to-firm levels. Journal of Financial Services Marketing.

[CR148] Suariedewi IGAAM, Suprapti NWS (2020). Effect of mobile service quality to e-trust to develop e-satisfaction and e-loyalty mobile banking services. International Research Journal of Management, IT and Social Sciences.

[CR149] Tabrani M, Amin M, Nizam A (2018). Trust, commitment, customer intimacy and customer loyalty in Islamic banking relationships. International Journal of Bank Marketing.

[CR150] Tam JLM (2019). Examining the role of customer self-efficacy in service encounters. Services Marketing Quarterly.

[CR151] Taylor SA, Donovan LAN, Ishida C (2014). Consumer trust and satisfaction in the formation of consumer loyalty intentions in transactional exchange: The case of a mass discount retailer. Journal of Relationship Marketing.

[CR152] Thakur R (2018). The role of self-efficacy and customer satisfaction in driving loyalty to the mobile shopping application. International Journal of Retail and Distribution Management.

[CR153] Tsao WC, Hsieh MT (2012). Exploring how relationship quality influences positive eWOM: The importance of customer commitment. Total Quality Management and Business Excellence.

[CR154] Tse DK, Wilton PC (1988). Models of consumer satisfaction formation: An extension. Journal of Marketing Research.

[CR155] Udo-Imeh PT, Awara NF, Essien EE (2015). Personality and consumer behavior: A review. European Journal of Business and Management.

[CR156] van der Aa Z, Bloemer J, Henseler J (2015). Using customer contact centres as relationship marketing instruments. Services Business.

[CR157] Vargo SL, Lusch RF (2008). Service-dominant logic: Continuing the evolution. Journal of the Academy of Marketing Science.

[CR158] Vater A, Schröder-Abé M (2015). Explaining the link between personality and relationship satisfaction: Emotion regulation and interpersonal behavior in conflict discussions. European Journal of Personality.

[CR159] Vazquez-Carrasco R, Foxall GR (2006). Influence of personality traits on satisfaction, perception of relational benefits, and loyalty in a personal service context. Journal of Retailing and Consumer Service.

[CR160] Veloutsou C (2015). Brand evaluation, satisfaction and trust as predictors of brand loyalty: The mediator-moderator effect of brand relationships. Journal of Consumer Marketing.

[CR161] Viviani JL, Komura A, Suzuki K (2021). Integrating dynamic segmentation and portfolio theories for better customer portfolio performance. Journal of Strategic Marketing.

[CR162] Wang S, Li Y, Tu Y (2018). Linking proactive personality to life satisfaction in the Chinese context: The mediation of interpersonal trust and moderation of positive reciprocity beliefs. Journal of Happiness Studies.

[CR163] Webber SS, Payne SC, Taylor AB (2012). Personality and trust fosters service quality. Journal of Business and Psychology.

[CR164] Weidmann R, Ledermann T, Grob A (2017). The interdependence of personality and satisfaction in couples. European Psychologist.

[CR165] Weiner IB, Greene RL (2008). Handbook of Personality Assessment.

[CR166] Wirtz J, Lovelock C (2016). Services marketing—People, technology, strategy.

[CR167] Yang Z, Peterson RT (2004). Customer perceived value, satisfaction, and loyalty: The role of switching costs. Psychology and Marketing.

[CR168] Yildiz E (2017). Effects of service quality on customer satisfaction, trust, customer loyalty and word of mouth: An application on cargo companies in Gumushane. Global Journal of Economics and Business.

[CR169] Yiu, E. (2018) One in seven Hongkongers is a millionaire. *South China Morning Post*. Accessed March 22, 2018. https://www.scmp.com/business/banking-finance/article/2138331/one-seven-hong-kong-millionaire-property-and-stock-market.

[CR170] Yun W, Hanson N (2020). Weathering consumer pricing sensitivity: The importance of customer contact and personalized services in the financial services industry. Journal of Retailing and Consumer Services.

[CR171] Zhang T, Tao D, Qu X, Zhang X, Zeng J, Zhu H, Zhu H (2020). Automated vehicle acceptance in China: Social influence and initial trust are key determinants. Transportation Research Part c: Emerging Technologies.

[CR172] Zhang, X., and S. Liu. 2021. Understanding relationship commitment and continuous knowledge sharing in online health communities: a social exchange perspective. *Journal of Knowledge Management *Vol. ahead-of-print No. ahead-of-print. 10.1108/JKM-12-2020-0883

[CR173] Zillig LMP, Hemenover SH, Dienstbier RA (2002). What do we assess when we assess a Big 5 trait? A content analysis of the affective, behavioral, and cognitive processes represented in Big 5 personality inventories. Personality and Social Psychology Bulletin.

